# Editorial Notes

**Published:** 1882-08

**Authors:** 


### EDITORIAL NOTES.

The Chautauqua Co. Medical Society, at its annual meeting, passed resolutions of protest against the action of the State Society on ethics, and instructed their delegates to vote to have it rescinded.

The *Scientific American* of July 29th contains an interesting article on the manufacture of opium, with illustrations of the various processes which the drug undergoes before ready for the market.

*The Medical Chronicle* is the latest addition to the ever changing list of medical journals. The record of births and deaths is about equal. Dr. George H. Rohe brings this last one into the world as a 24-page monthly, published in Baltimore. Success to it.

THE strictures of the *London Lancet* on the unsanitary condition of Brighton (England's great health resort) have been met by the town authorities with a suit for libel. The statement is made that a fund of $30,000 is guaranteed to defend the reputation of the place as a health resort.

THE circulation of the JOURNAL has been largely increased of late, evidence that our editorial efforts are appreciated. We not only ask for new subscribers but prompt payment of subscriptions upon the part of the old ones. Bills will be presented, for which we request prompt and favorable attention.

THE working bulletins issued by Parke, Davis & Co., giving practical observations on the action of their medicinal agents, is a movement in the right direction and should receive encouragement from the profession. It gives the specific action of remedies from a variety of sources and from the experience of different observers.

OUR congratulations are due our much-esteemed friend, Dr. Ed. Storck, of this city, for his recovery from the dangerous illness with which he has been afflicted for several months. We hope renewed health and strength will be given him for future work in the profession in which he has labored so earnestly and successfully in the past.

DYSPEPSYN.—Some months ago we acknowledged the receipt of a quantity of this preparation, which the manufacturers wished us to make a trial of. Our experience with it so far has abundantly sustained the high claims made for it. It is one of the best remedies in the cases which require artificial aid to facilitate digestion. It is superior to the ordinary pepsins and is much less expensive to the patient.

WE have received a notification from the Secretary of the National Board of Health, stating that insufficient provision having been made in the "Sundry Civil Appropriation Bill" for the present year for the expenses of the Board, the publication of the *Bulletin* would be at once suspended, should the bill, as reported, pass the House. We hope it is not too late to obtain the necessary legislation to ensure the continuance of this valuable publication. Its discontinuance would be a public loss.

